# Neural activations associated with feedback and retrieval success

**DOI:** 10.1038/s41539-017-0013-6

**Published:** 2017-11-02

**Authors:** Carola Wiklund-Hörnqvist, Micael Andersson, Bert Jonsson, Lars Nyberg

**Affiliations:** 1 Department of Psychology, Umeå University, Umeå, Sweden; 2 Umeå Center for Function Brain Imaging (UFBI), Umeå University, Umeå, Sweden; 3 Department of Integrative Medical Biology, Umeå University, Umeå, Sweden; 4 Department of Radiation Sciences, Umeå University, Umeå, Sweden

## Abstract

There is substantial behavioral evidence for a phenomenon commonly called “*the testing effect*”, i.e. superior memory performance after repeated testing compared to re-study of to-be-learned materials. However, considerably less is known about the underlying neuro-cognitive processes that are involved in the initial testing phase, and thus underlies the actual testing effect. Here, we investigated functional brain activity related to test-enhanced learning with feedback. Subjects learned foreign vocabulary across three consecutive tests with correct-answer feedback. Functional brain-activity responses were analyzed in relation to retrieval and feedback events, respectively. Results revealed up-regulated activity in fronto-striatal regions during the first successful retrieval, followed by a marked reduction in activity as a function of improved learning. Whereas feedback improved behavioral performance across consecutive tests, feedback had a negligable role after the first successful retrieval for functional brain-activity modulations. It is suggested that the beneficial effects of test-enhanced learning is regulated by feedback-induced updating of memory representations, mediated via the striatum, that might underlie the stabilization of memory commonly seen in behavioral studies of the testing effect.

## Neuroscience: Test-enhanced learning with feedback

Repeated testing with feedback shapes functional brain activity and strengthens new learning. A team led by Carola Wiklund-Hörnqvist at Umeå University, tracked brain activity in humans while being repeatedly tested with or without feedback. Higher hippocampal activity was found for feedback compared to no feedback, and learning from feedback was related to increased activity predominantly in the insula. For retrieval success, up-regulated activity in fronto-striatal regions was evident at the first successful retrieval with a marked decrease across consecutive tests. Those results indicate that while both feedback and retrieval success are key aspects that foster the “testing effect”; they operate at different functional levels in the brain. Future studies are needed to further explore the efficiency of test-enhanced learning in relation to individual variation in cognitive proficiency.

## Introduction

A substantial number of behavioral studies have demonstrated that repeated testing enhances learning and retention of to-be-learned material more than restudy, a phenomenon known as the *testing effect*.^1–3^ This effect has been observed after shorter, as well as longer retention intervals. Cognitive theories suggest that the mnemonic benefits of test-enhanced learning can be attributed to enhanced memory strength,^4–6^ and that feedback is a critical factor.^7–10^ Feedback with the correct answer improves learning as the additional study opportunity prevents retrieval failures and errors from being repeated,^8,11^ and supports maintaining correct responses over consecutive tests.^12^ However, despite substantial behavioral evidence for a key role of feedback, little is known about the underlying neuro-cognitive processes for feedback effects on test-enhanced learning (see ref. 13 for a review).

It has been suggested that testing strengthens memory either by “filtering out” irrelevant representations during retrieval practice^6^ or by updating contextual representations.^14^ In a related vein, neurocomputational models^15,16^ suggest that updating of task-relevant information is related to fronto-striatal interactions, where the striatum acts as a gating mechanism that regulates input of task-relevant information to the prefrontal cortex (PFC). More recently it has been suggested that the same fronto-striatal mechanism supports the selection of maintained representations in PFC that will be successfully retrieved (i.e. output gating,^17,18^). Thus, in line with psychological explanations for the testing effect, striatum could critically account for how initial testing strengthens memory by supporting selective updating and subsequent selection of appropriate responses available within WM during effortful retrieval.^17,19,20^

Abundant research has stressed the importance of striatal activity during declarative retrieval success,^20–25^ with striatal activity increasing as a function of increased task difficulty,^24,26,27^ but with a rapid decrease over the course of successful learning.^28^ Moreover, neuroimaging studies have provided evidence for the ventral striatum (VS) as important in tracking positive outcomes following a modified retrieval strategy,^29^ suggesting that new learning after feedback (i.e. shifting from retrieval failure to success) might be strengthened by striatal recruitment during subsequent successful retrieval.^30^

Few imaging studies have investigated the testing effect (see ref. 13 for a review), but select studies have reported evidence for striatum being more active during testing compared to study,^31,32^ but none reported learning-related changes across consecutive tests. Here, we hypothesized that one potential mechanism by which repeated testing with feedback strengthens memory is via fronto-striatal interactions.^20^

We used an event-related fMRI paradigm to track neural responses during repeated testing with correct answer feedback. We scanned young healthy participants while they were learning Swahili–Swedish word-pairs across three consecutive cued-recall tests with feedback (hereafter denoted as T1, T2, and T3). Data were analyzed in relation to both the feedback event and the retrieval event across repetitions (see section “Methods” for details). To minimize expectancy feedback, test items with feedback [correct target word] were uniquely interspersed with test items without feedback [¤¤¤]. We expected higher activity in the hippocampus^27,33^ for feedback compared to no feedback, and, in line with fMRI studies examining the potentiated effects of testing on successful re-encoding,^34,35^ higher activity in insula following a retrieval failure compared to a retrieval success when feedback was present. Moreover, to the extent that feedback has a positive impact on learning,^7–11^ our primary aim was to investigate its effect upon subsequent retrieval trials.^12^ In line with prior research, we expected higher striatal activity for successfully retrieved items compared to failures^20–22,25^ and reduced fronto-striatal activity as a function of consecutive successful retrievals, likely reflecting less need for executive control functions.^24,26,27^

## Results

### Behavioral results

#### Repeated cued recall testing with FB

Mean proportion of response correctness for items judged as “know and correct” at T1 was 0.34 (*SE* = 0.05), at T2, *M* = 0.61 (*SE* = 0.06), and at T3, *M* = 0.69 (*SE* = 0.06) indicating that feedback significantly enhanced learning across the three consecutive tests. A one-way repeated ANOVA showed a significant learning effect across the three repetitions for items judged as “know and correct” [*F*(2,42) = 87.28, *MSE* = 0.08, *p* < 0.001, *n* ^2^_p_ = 0.81], and pairwise comparisons confirmed a significant improvement over each consecutive test (between T1 and T2, *p* < 0.001 and between T2 and T3, *p* = 0.003). The participants did not have a sufficient number of trials (for the purpose of fMRI analyses) that were unsuccessfully retrieved at T1 and T2, but successfully recalled at T3, so T3 was therefore not included in the fMRI analysis.

### fMRI results

#### Retrieval success

**Fig. 1:**
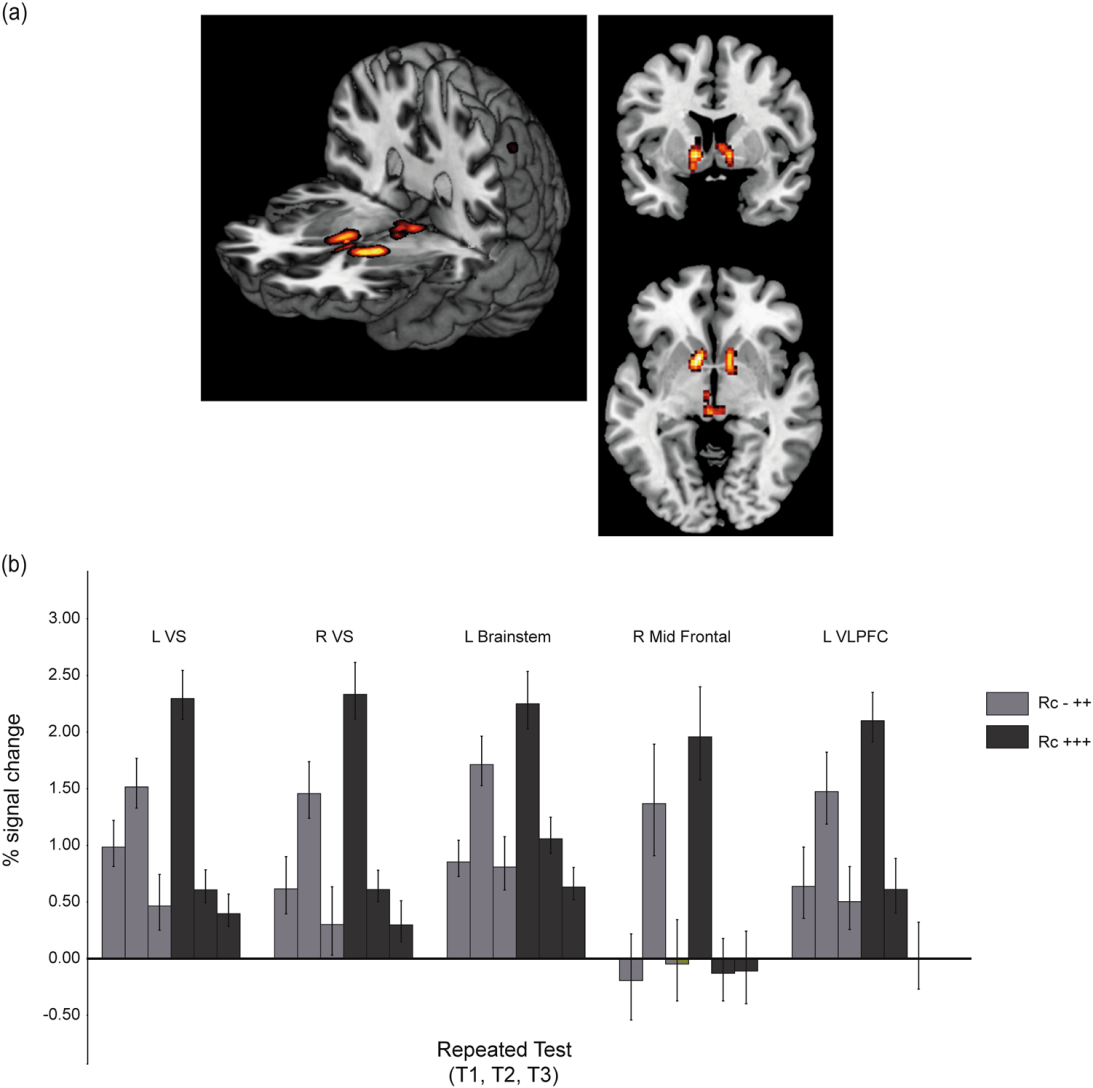
**a**, **b** The significant interaction effect for the retrieval event. **a** Brain regions showing different activation during the retrieval event related to retrieval success in the left and the right hemisphere: bilateral VS [−10 10 −4; 14 2 −8] and brainstem [−2 −26 −4]. **b** Signal change in the BOLD signal during feedback related to retrieval success. Error bars denote ±1 s.e.m. *Note:* The third test (T3) was not included in the 2 × 2 ANOVA, but for illustrative purposes the T3 results are illustrated in Fig. 2b

A 2 (retrieval outcome; success versus failure) × 2 (test; T1, T2) ANOVA was conducted. There was a significant retrieval outcome by test interaction, [*F* (1, 72)* = *26.91, *p* < 0.05 (family-wise error (FWE))], with the strongest effect in the bilateral VS (see Fig. 1a, b). Significantly elevated activity was seen at the first successful retrieval (at T1 or T2) in bilateral VS [−10 10 −4 and 14 2 −8], in a right mid-frontal region [46 54 18], left inferior frontal triangularis [-46 26 20], the left brainstem [mesencephalon, −2 −26 −4], and two small clusters in left parietal cortex [−46 −52 46] extending into left angular gyrus [−36 −50 36]. As can be seen in Fig. 1a, b, for items successfully retrieved on T1, as well as T2 and T3 (Rc+++), these regions were strongly active at T1 along with subsequent activity reduction on T2 and T3. By contrast, for items unsuccessfully retrieved at T1 but successfully retrieved at T2 and T3 (Rc-++), the reversed pattern was evident with an activity increase from T1 to T2 followed by a marked reduction from T2 to T3. Notably, while this effect may partly be attributed to a retrieval success effect per se, the influence of feedback critically affected the change in outcome from T1 to T2 for the Rc-++ items.

#### Feedback

To identify regions associated with feedback per se, we contrasted all events associated with feedback [correct target word] versus no feedback [¤¤¤] independently of response correctness at T1. Feedback elicited greater activity than no feedback in the left hippocampus (HC, BA 28), inferior frontal gyrus (IFG, BA 44 and 47), middle/superior temporal lobe (BA 21 and 37), inferior/superior parietal lobe, and bilateral insula (BA 48; see Table 1). The reversed comparison, no feedback > feedback, yielded no clusters significant at the statistical threshold.

**Table 1:** Regional responses during the feedback event contrasting FB > no FB at T1

Cluster	Local maxima	Hem	BA	*x*	*y*	*z*	*Z*-score	Voxels (*k*)
1	Hippocampus	L	28	−16	−8	−18	5.09	76
	Parahippocampus	L	34					
	Amygdala	L	35					
2	Inferior frontal opercularis	L	44	−60	10	20	5.01	808
	Precentral	L	4	−52	10	14	4.54	
	Inferior frontal opercularis	L	6	−42	6	24	4.46	
	Inferior frontal triangularis	L	44	−54	10	26	4.33	
	Opercularis rolandic	L	48	−38	14	28	4.27	
3	Superior occipital	L	7	−28	−66	40	4.24	877
	Middle occipital	L	19	−46	−44	46	4.24	
	Superior parietal	L	39	−26	−72	28	3.96	
	Inferior parietal	L	40	−38	−42	38	3.88	
4	Fusiform	L	20	−42	−62	−16	4.28	445
	Inferior temporal	L	37	−56	−62	−4	4.13	
5	Inferior frontal triangularis	L	45	−52	38	6	4.38	299
	Middle frontal	L	45	−46	34	12	4.36	
	Inferior frontal triangularis	L	47	−52	32	−2	4.05	
	Inferior frontal orbital	L	48	−42	42	16	3.90	
6	Insula	L	48	−36	14	2	4.32	102
7	Insula	R	48	38	16	−2	4.30	51
8	Supplementary motor area	L	NA	0	8	58	4.29	159
	Supplementary motor area	R	6					
9	Middle temporal	L	21	−56	−32	−6	4.22	130
10	Middle temporal	L	21	−50	−46	2	4.07	32

*Note*: Up to five local maxima are reported for each cluster. Coordinates (*x*,* y*, *z*) in MNI space (SPM12). *Z*-values at the peak voxel. Voxel: *p* < 0.01 (FDR). Cluster: *k* ≥ 20

*Hem*  hemisphere, *BA*  Brodmann area

**Fig. 2:**
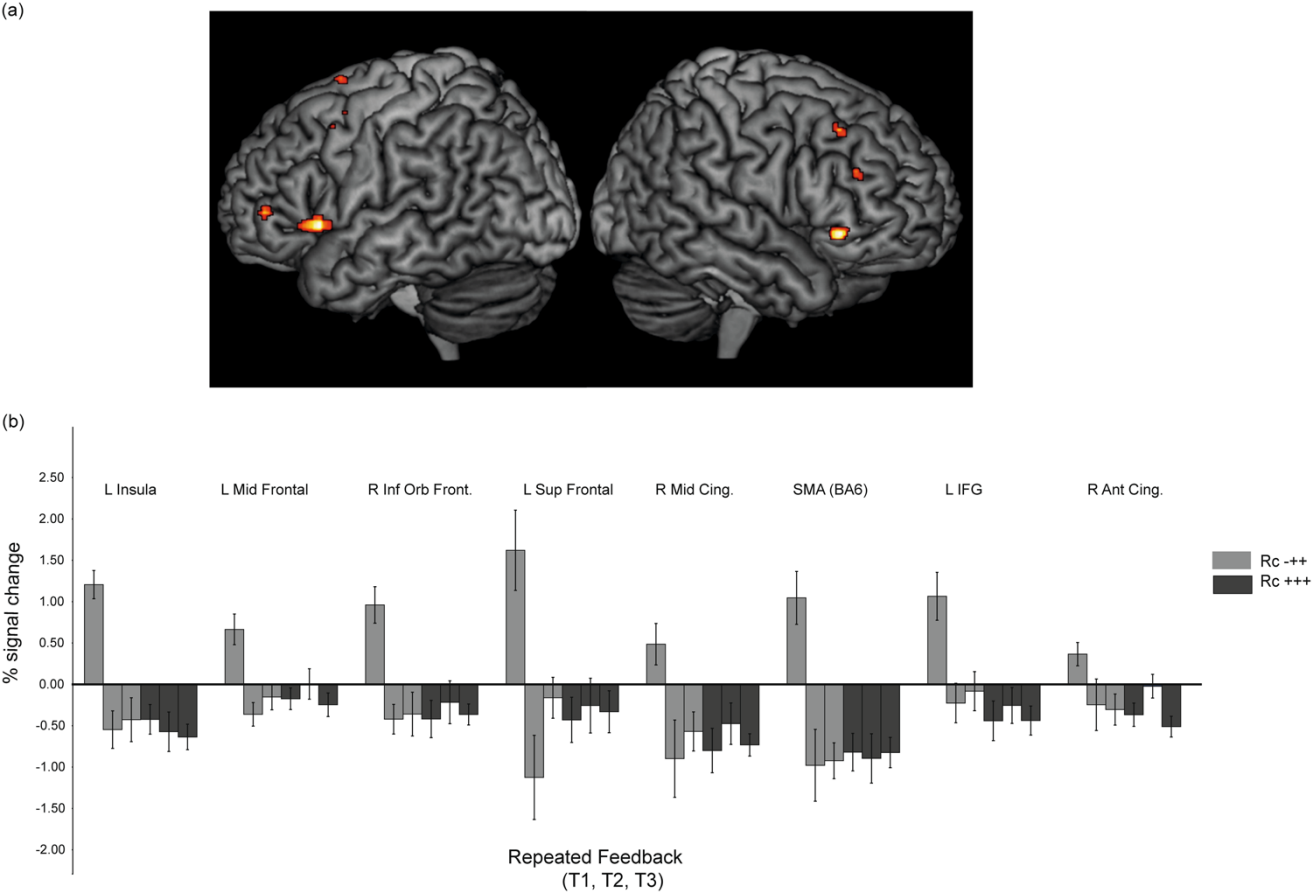
**a**, **b** The interaction effect for the feedback event. **a** Brain regions showing differential activation during feedback related to prior retrieval success in the left and the right hemisphere. **b** Change in the BOLD signal during feedback related to retrieval success. Error bars denote ±1 s.e.m. The activations were identified at a more lenient statistical threshold (*p* < 0.001 uncorrected at the voxel level, *k* ≥ 5). *Note:* The third test (T3) was not included in the 2 × 2 ANOVA, but for illustrative purposes the T3 results are illustrated in Fig. 3b

The critical comparison concerns differences in brain activity at the feedback event in relation to retrieval success (Rc-++ versus Rc+++). A 2 (retrieval outcome; success versus failure) × 2 (test; T1, T2) ANOVA was conducted to examine whether the BOLD signal change during feedback was related to subsequent retrieval success in T2. No significant interaction effects above the predefined statistical threshold were found, but at a more lenient threshold (*p* < 0.001 uncorrected at the voxel level, *k* ≥ 5), significantly higher activity at T1 was found for Rc-++ items in several bilateral frontal regions compared to Rc+++ items (see Fig. 2a, b). Thus, following retrieval failure, activity in several PFC regions (but not VS) was markedly elevated at the time of feedback, possibly reflecting re-encoding, whereas no such effect was seen at the feedback event following successful retrieval.

## Discussion

Compared with the extensive behavioral evidence for test-enhanced learning as superior to a study only condition, and feedback as critical for the effect, little is known about the neuro-cognitive processes underlying these behavioral outcomes. Here we used fMRI to track brain responses during repeated testing with feedback. The first goal of the study was to investigate brain regions in relation to feedback. Feedback may be seen as a new learning opportunity. In line with that notion, increased hippocampal activity was found for feedback compared to no feedback, and learning from feedback was related to increased activity in the insula. A second goal was to examine how the retrieval activity was modulated by feedback. We found strong VS activity during the first successful retrieval, but marked decrease over consecutive tests. Thus, we provide novel evidence that feedback strengthens test-enhanced learning, predominantly via the VS.

While some prior imaging studies, explicitly investigating the testing effect, have found the striatum to be more strongly activated during testing compared to study,^31,32^ we here extend those findings by showing that striatal recruitment during testing is specifically related to retrieval success. A similar activity pattern across consecutive tests was seen in bilateral frontal regions and in a region within the brainstem. Higher activity in these regions was only found at the first retrieval success trial, independently of when it occurred (T1 or T2), and the response showed a marked decrease as a function of subsequent successful retrieval trials.

Whereas our VS regions fell within the retrieval success network identified in a recent meta-analysis,^25^ the modulation of the VS response can also be understood in terms of cognitive demands.^24,27^ For example, striatum along with PFC supports cognitive-control mechanisms needed during declarative memory retrieval, and the modulation of fronto-striatal activity is positively related to the degree of cognitive effort needed to successfully retrieve the target.^20,24,26,27^ Mimicking our results, the ability to correctly respond during high memory load has been associated with the right middle frontal region found in our study.^36^ As for the left frontal region, a multimodal study combining fMRI and SPECT provided evidence for that activity in left VLPFC was positively associated with striatal D2 binding during updating of long-term memory representations.^23^ The latter finding corresponds well with a recent high-resolution fMRI study in which brainstem dopamine phasic signals mediated updating of contextual representations into working memory.^37^ As indicated by our significant retrieval success interaction effect, we also found evidence for a region within the brainstem that followed the same activity pattern as the fronto-striatal regions. D’Ardenne and colleagues^37^ used high-resolution fMRI, and the location of our brainstem region seemed to overlap with their observations (see also refs. 38–40 for related reports).

There was a marked decrease in the BOLD response from the first to the second successful retrieval trial. We suggest that one possible explanation for this pattern of results is related to memory demands during selective updating of contextual representations during the learning phase. At T1, no feedback had been introduced and more cognitive control was needed to update potential candidates that will be selected and subsequently retrieved. At T2, this updating procedure was less demanding since irrelevant representations had been “filtered out” at T1. When the retrieval attempt at T1 failed, but became successful at T2, this led to updating of the relevant representations. Subsequently, as indicated in Fig. 1b, less updating processes were needed at later successful retrieval trials. This account corresponds well with the cognitive explanations of how test-enhanced learning strengthens memory across consecutive tests.^6,14^ Further evidence for this notion comes from both neurocomputational models^15,16,41^ and neuroimaging studies, which have provided evidence for an interplay between midbrain, frontal, and striatal regions during selective updating of relevant information into working memory^37,42^ and long-term memory.^43^

We know from a wealth of literature that testing is not a neutral event, but an active process that changes memory. The basic idea with test-enhanced learning—“test yourself during initial learning”—implies that the knowledge level is generally low, and hence that the inclusion of feedback is a critical factor to support improvement in learning. Direct comparison of feedback versus no feedback revealed elevated activity for feedback in mainly left lateralized regions in the hippocampus and fronto-temporo-parietal cortical regions, which extend previous studies.^27,33^ In contrast to behavioral studies,^12^ which have implied that feedback strengthens initial correct responses accompanied by low confidence, the current study failed to demonstrate such an effect. Instead, feedback played little, if any, role after the first successful retrieval. One explanation for the inconsistent findings might be related to the fact that we only included “know and correct” responses (i.e. high-confident responses), as too few items received “believe and correct” responses (i.e. low-confident responses). In addition, our results are in agreement with those of Pashler and colleagues,^8^ which showed that even if feedback more than doubled retention compared to test with no feedback, feedback following a correct response made little difference independent of response confidence.

Despite substantial evidence for the VS as important during feedback,^44^ no differential VS activation was found at the time of the feedback event. Feedback in our paradigm was defined as the correct target word (not positive versus negative feedback), and thus more likely serving as a re-encoding event rather than reward per se. In line with this reasoning, and in agreement with our findings of insula activity change during feedback following retrieval failures, neuroimaging studies investigating the potentiated effects of testing on successful re-encoding have provided evidence for insular activation during re-study,^34,35^ with higher activity following a retrieval failure.^34^

Whereas a substantial number of studies have demonstrated that test-enhanced learning is superior compared to a diversity of other pedagogical methods,^45^ few studies have investigated its efficiency in relation to individual differences in cognitive proficiency (e.g. working memory^46,47^). It has been shown that test-enhanced learning might be equally beneficial independent of individual variations in working memory capacity^47^ and even recognized as especially beneficial for those with lower working memory capacity.^46^ These results are of educational significance, and consistent with the results of the current study showing that test-enhanced learning taxes executive processes early in the learning phase but less executively demanding so as a function of improved learning. Future studies are needed to further explore the efficiency of test-enhanced learning in relation to individual variations in cognitive proficiency. This is an important issue given that we want to transform the science of learning into educational practice.

In conclusion, the present findings suggest that feedback strengthens test-enhanced learning via the striatum.

## Materials and Methods

### Ethics statement

In accordance with the Helsinki declaration, written informed consent was obtained from each participant prior to the start of the study. Methods were performed in accordance with relevant regulations and recommendations, and the study was approved by the Regional Ethics Committee at Umeå University.

### Participants

Twenty-two neurologically healthy subjects (mean age 26.2 years; age range, 21–33 years, 7 males) participated in the study. They were all right handed by self-report, and they all had normal or corrected-to-normal vision. All were Swedish natives, and no participant reported prior experience with the Swahili language. Participants received 300 Swedish kronor for their participation. Data from two participants were excluded from two of the analyses as they had fewer than five events in the item categories examined. Data from additional one participant were discarded from all analyses due to registration failures.

### Study overview

The study included a pre-scan study phase followed by a fMRI session focusing on repeated testing with feedback (three times: T1, T2, and T3). Critically, to be able to relate the outcome from the current study design to the traditional testing effect (Test versus Study), we collected behavioral data from an additional sample of subjects (*n* = 39) that completed the same learning paradigm, by comparing a repeated testing with feedback condition with a repeated study condition. Importantly, the results confirmed a typical testing effect one week after the intervention (*p* < 0.01).

### Material and experimental set up

The to-be-learned material was 60 Swahili–Swedish word-pairs previously used.^48^ Half of the word-pairs were tested once without feedback [¤¤¤], and the other half was tested three repeated times with correct answer feedback [correct target word]. In order to minimize the expectancy for feedback (since they were presented three times), 15 of the 30 word-pairs without feedback were interspersed within the test list a second time to make the occurrence of feedback more unpredictable. Importantly, the second presentation of each of these extra words without feedback were excluded from the analysis. For each test list and subject, word-pairs tested with and without feedback were uniquely interleaved and the items uniquely randomized for each subject. The lag between each repetition of a word ranged from 4 to 27 items. The main purpose with the no feedback condition was: (1) to prevent the influence of expectancy feedback to occur and (2) to serve as a baseline for identifying regions associated with feedback [correct target word] as compared to no feedback [¤¤¤] commonly used within the behavioral testing effect literature. In the analyses, items tested without feedback at T1 served as a baseline comparison.

### Procedure

#### Pre-scan study phase

Each participant studied the 60 word-pairs in front of a computer five consecutive times. Each word-pair was randomly presented one-by-one in the middle of the screen at a rate of 5 s each. The Swahili word was presented on the left side, and the Swedish word on the right side. The participants were instructed to try to memorize the word-pairs. They did not receive any information about the following test procedure in the scanner. The pre-scan study phase took ∼25 min. Immediately after the pre-study phase was completed they got instructions about the test procedure in the subsequent fMRI session. This was followed by practice a procedural familiarization task using nine common Swedish–English word-pairs to ensure they understood the testing procedure in the scanner (see ref. 48 for a similar procedure).

#### fMRI session

**Fig. 3:**
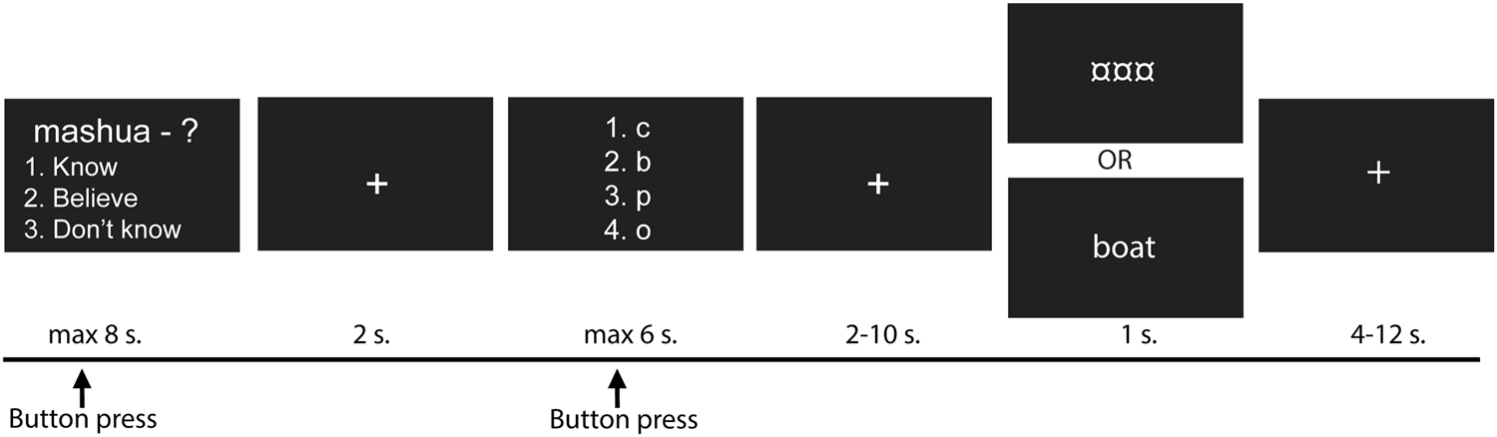
Schematic overview of the task. Each word-pair was repeatedly tested three times with correct-answer feedback. First, the Swahili word appeared on the screen and subjects were asked to respond whether they could recall the Swedish counterpart or not. Next, they had to respond the second letter in the Swedish word, followed by either feedback or no feedback. To prevent expectancy feedback, test items with feedback were uniquely interspersed with test items without feedback

During the scanning session, half of the items were tested three times with feedback (T1, T2, and T3) and the other half was tested once/twice without feedback (see Fig. 3). All items were uniquely interspersed within each test list and the specific items uniquely randomized for each subject.

The experimental paradigm used was partially the same as in our previous study^48^ but now included a feedback/no feedback event. Participants received the Swahili word as a probe and were asked to recall the Swedish counterpart. The Swahili word was shown for a maximum of 8 s. Within this time, participants were asked to respond by pressing a four-button keypad with their right hand fingers to indicate whether they had recalled a Swedish word they (1) “Knew was correct” (index finger), (2) “Believed was correct” (middle finger), or (3) “Did not retrieve a word” (ring finger). Next, a fixed crosshair (2 s) appeared on the screen. Participants were then asked to choose among four alternatives for the second letter in the Swedish word they had just retrieved, using the right hand fingers within 6 s. The position of the correct answer relative to the lures was systematically varied, such that the target appeared equally often in each of the four possible positions across words, as well as varied across repetitions. A jittered crosshair appeared on the screen (2–10 s) followed by either feedback or no feedback for 1 s. Feedback was presented in the form of the correct target word [boat], whereas in the no feedback condition, the feedback event was replaced by three symbols [¤¤¤], keeping the time for each test trial constant (see Fig. 3). Independent of feedback or no feedback, it was always presented in the middle of the screen. Immediately after feedback/no feedback presentation, a jittered crosshair appeared on the screen (4–12 s) before the next trial begun. The scan session ended with structural imaging. In total, the scanning session lasted for ~1½ h.

### Item classification

Word-pairs stated as “know is correct” and given the correct second letter was considered as “retrieved items”, whereas word-pairs stated as “did not retrieve a word” regardless of the second letter were considered as “retrieval failures”. All word-pairs (items) repeatedly tested with feedback were post-hoc sorted as follows: (1) Items correctly recalled three times (Rc+++) and (2) Items unsuccessfully retrieved at T1, but correctly recalled at T2 and T3 (Rc-++). Items responded as “believed was correct” was excluded from all analyses.

### fMRI data acquisition and preprocessing

All images were acquired using a 3.0 T whole-body MRI system (MR 750, GE Medical Systems) equipped with a 32 channels head coil. The scanner parameters were identical to those used in our previous study.^48^ T2* weighted images were obtained with a single-shot GE-EPI sequence used for BOLD imaging. The sequence had the following parameters: echo time 30 ms; repetition time 2000 ms; flip angle 80°; FOV 250 × 250 mm; matrix 96 × 96, and slice thickness 3.4 mm (37 slices acquired). Ten dummy scans were collected before the data collection started to allow for equilibration of the fMRI signal. High-resolution T1-weighted structural images were obtained for each participant. Stimuli were presented on a computer screen seen by the participant through a mirror attached to the head coil. Stimuli presentation was handled by a PC running E-prime version 2.0 (Psychology Software Tools) and Lumitouch fMRI optical response keypads (Photo Control) were used to collect responses. Scanner noise was reduced with headphones and earplugs, and cushions in the coil reduced head movement.

In preprocessing and analyzing the data SPM12 (The Wellcome Department of Cognitive Neurology, Institute of Neurology, University College London, London, UK) was used, with a batch function in an in-house program (Data Z). The preprocessing included correction for slice timing and movement, and coregistering to each subjects anatomical T1-image. The T1-images were segmented and a dartel-template with corresponding deformation field-files were created (DARTEL^49^). These were used for normalizing the fMRI-images to MNI-space. They were then smoothed (spatially low-pass filtered with an 8 mm FWHM Gaussian filter kernel). Statistical analyses were calculated on the smoothed data with a high-pass filter (128 s cutoff period) to remove low-frequency noise.

### fMRI data analyses

Statistical analyses were performed in two stages. In the first stage, and for each subject, we created two separate general linear models (GLMs) at T1: one for the retrieval event and one for the feedback event. The GLM for the retrieval event contained two regressors: word-pairs with subsequent feedback and word-pairs with no subsequent feedback. The GLM for the feedback event contained two regressors: feedback and no feedback. We also included nuisance regressors for the six movement parameters. Initial whole brain analyses were conducted to directly test for differences between the Test with feedback and Test without feedback condition, both at the retrieval event and the feedback event, respectively, at T1. Those analyses aimed to confirm that (1) no differences were evident during retrieval at T1, and (2) identified regions uniquely involved in feedback as compared to no feedback. The statistical maps for those initial analyses were considered as statistically reliable, if they passed a false-discovery rate (FDR) corrected threshold of *p* < 0.01 and *k* ≥ 20 at the cluster level.^50^

In the second stage, for each subject, a GLM was set up in SPM consisting of regressors for Rc-++ and Rc+++ at both retrieval and feedback, separate for each repetition (T1 and T2). In total, we had 12 regressors of interest. We also had nuisance regressors for multiple choice (i.e. second letter alternatives) and the six movement parameters. Each event (retrieval and feedback) were modeled by separate stick functions corresponding to the onset of stimulus presentation convolved with the canonical hemodynamic response function (HRF). We run the data analysis both with and without the time derivative of the HRF included, and the results were comparable with either approach.

To address the main questions of the study: (1) what brain regions are uniquely involved during repeated testing and how are they related to retrieval success, and (2) what brain regions are uniquely recruited during feedback and how are they related to retrieval success, two separate ANOVA:s were performed. First, to examine how retrieval affects functional brain activity across repetitions related to retrieval success, we performed a 2 (retrieval success; Rc-++, Rc+++) × 2 (repetition; T1, T2) ANOVA at the retrieval event. Next, to examine how feedback affects functional brain activity across repetitions related to retrieval success, we performed a 2 (retrieval success; Rc-++, Rc+++) × 2 (repetition; T1, T2) ANOVA during the feedback/no feedback presentation. These analyses aimed to test for interaction effects. Unless otherwise stated, the statistical voxel threshold for the interaction effects was set to *p < *0.05, FWE corrected and a cluster threshold *k* ≥ 5 voxels. Data from two subjects were discarded from the two ANOVAs, as fewer than five items per repetition in each condition of interest were present.

The sample size was determined based on prior behavioral studies, which have shown that ∼20 subjects are sufficient to reach significant behavioral results, and some published power analyses of fMRI studies, recommending that ∼15–20 subjects are needed for 80% power (e.g. ref. 51). Data are available from the corresponding author on request.
